# Identifying structures, processes, resources and needs of research ethics committees in Egypt

**DOI:** 10.1186/1472-6939-11-12

**Published:** 2010-06-28

**Authors:** Hany Sleem, Samer S El-Kamary, Henry J Silverman

**Affiliations:** 1 National Hepatology and Tropical Medicine Research Institute, Cairo, Egypt; 2 University of Maryland School of Medicine, Baltimore, Maryland, USA

## Abstract

**Background:**

Concerns have been expressed regarding the adequacy of ethics review systems in developing countries. Limited data are available regarding the structural and functional status of Research Ethics Committees (RECs) in the Middle East. The purpose of this study was to survey the existing RECs in Egypt to better understand their functioning status, perceived resource needs, and challenges.

**Methods:**

We distributed a self-administered survey tool to Egyptian RECs to collect information on the following domains: general characteristics of the REC, membership composition, ethics training, workload, process of ethics review, perceived challenges to effective functioning, and financial and material resources. We used basic descriptive statistics to evaluate the quantitative data.

**Results:**

We obtained responses from 67% (12/18) of the identified RECs. Most RECs (10/12) have standard operating procedures and many (7/12) have established policies to manage conflicts of interests. The average membership was 10.3 with a range from 7-19. The predominant member type was physicians (69.5% of all of the REC members) with little lay representation (13.7%). Most RECs met at least once/month and the average number of protocols reviewed per meeting was 3.8 with a range from 1-10. Almost three-quarters of the members from all of the 12 RECs indicated they received some formal training in ethics. Regarding resources, roughly half of the RECs have dedicated capital equipment (e.g., meeting room, computers, office furniture, etc); none of the RECs have a formal operating budget. Perceived challenges included the absence of national research ethics guidelines and national standards for RECs and lack of ongoing training of its members in research ethics.

**Conclusion:**

Our study documents several areas of strengths and areas for improvements in the operations of Egyptian RECs. Regarding strengths, many of the existing RECs meet frequently, have a majority of members with prior training in research ethics, and have written policies. Regarding areas for improvements, many RECs should strive for a more diverse membership and should receive more financial resources and administrative support personnel. We recommend that RECs include more individuals from the community and develop a continuing educational program for its members. Institutional officials should be aware of the resource capacity needs of their RECs.

## Background

Research involving human subjects has increased in various regions in the developing world, including the Middle East [1]. Concerns, however, have been expressed that the intensification of research activities has not been accompanied by a corresponding increase in institutional research ethics capacity, including functioning ethics review systems [2-4]. Several studies have demonstrated insufficient ethics capacity among investigators from the different countries of the Eastern Mediterranean region [5,6]. In general, commentators have voiced concerns that research ethics committees (RECs) in developing countries might not be able to promote high standards of human subject protection due to inadequate financial and material resources, lack of adequately trained REC members, insufficient diversity of membership, lack of REC independence, and inability to monitor approved protocols [2,7-9].

While the establishment of RECs in the Middle East has recently increased, the quality and consistency of ethical review remains unclear. Specifically, little data are available regarding processes of ethics review, member composition, training of members, workload and resource needs of RECs, and challenges that RECs encounter in this region. In contrast, two studies have identified the characteristics, resources and needs of RECs in Africa [10,11]. Documenting the extent of the functional status and capacity of existing RECs would be helpful in targeting areas for improvement and providing capacity building programs to meet the needs of these RECs. Such information would also help guide policy makers (e.g., Ministry of Health and top officials at academic institutions) and training programs in their support of strengthening of such committees. Accordingly, this survey study was undertaken to better understand the operating characteristics, status, and challenges of existing RECs in Egypt.

## Methods

### Study Design

A cross-sectional survey study design.

### Survey Tool

We adapted a survey tool used in a previous study assessing resources and needs of research ethics committees in Africa [10]. The completed survey tool contained the following domains of requested information: general characteristics of the REC, membership composition, ethics training, workload, process of ethics review, perceived challenges to effective functioning, and financial and material resources.

### Participants

Chairs or their designee of known existing RECs in Egypt.

### Recruitment and Distribution Process

We identified the existence of RECs in Egypt by conducting web searches, contacting REC members who attended research ethics workshops in Egypt, searching the list of registered RECs on the Office of Human Research Protections (OHRP) website, and contacting trainees of the Fogarty International Center/National Institutes of Health (FIC/NIH) who are members of Egyptian RECs [12].

We distributed the surveys to the chairpersons or their designees of identified RECs between late 2007 and early 2008. Follow-up telephone calls were made to all contacts to ensure they had received the survey. When requested, the Egyptian principal investigator (HS) would visit the chairperson to answer any questions about the survey. Each respondent REC was sent a confidential copy of its individual data superimposed on a summary result. This served to verify the analysis and enabled each respondent to compare his or her committee's responses relative to the whole sample.

### Statistical Analysis

We used basic descriptive statistics to present the quantitative data.

### Ethical Considerations

#### Informed Consent

A cover letter accompanied each survey containing information about the purpose, risks, benefits, confidentiality, contact information, and voluntary nature of the study.

#### Confidentiality

A code linking respondents to their surveys were kept separately in a secure, locked, location. Confidentiality was assured to enhance accurate reporting by the respondents. We assured respondents that only aggregate data would be described in public reports.

#### Ethics Review

This study was approved by the Research Ethics Committee at the National Hepatology and Tropical Medicine Research Institute, Cairo, Egypt and the Institutional Review Board at the University of Maryland, School of Medicine, Baltimore, USA.

## Results

We obtained responses from 67% (12/18) of the identified RECs. Of these RECs, 7 were situated in academic institutions, 4 in research institutions, and 1 in a non-governmental organization (NGO).

### General Characteristics

Table 1 shows the general characteristics of the respondent RECs. All RECs reported being involved in the ethical review of research protocols. There were variable responses regarding the specific types of researches that they reviewed; for example, master thesis, doctoral thesis, drug trials, and international studies. Most (10/12) reported having written standard operating procedures (SOPs), many (7/12) have established policies for disclosure and management of potential conflicts of interest, and half have a process whereby research participants can register a complaint. RECs were asked to rate the appropriateness of a list of international ethical guidelines for use in their review of research. Ratings were 'very appropriate', 'somewhat appropriate', 'not really appropriate', and 'not inappropriate'. Guidelines rated by RECs as being 'very appropriate' included the CIOMS guidelines (64%); the Declaration of Helsinki (78%), and the IOMS (56%). Only one REC rated the Belmont Report as "very appropriate". RECs were asked to whom they reported and 4 stated the Dean of their faculty, 4 reported to a top official or an official person or body of the University (President, Vice-President, Secretary General, or Faculty Council of the Institution), 1 reported to the Department Head, 1 reported to a high official of the NGO, and 2 stated 'none'.

**Table 1:** Characteristics of Research Ethics Committees (n = 12)

Characteristic	Frequency of RECs
Type of researches that are reviewed	
Master's thesis	67% (8/12)
PhD thesis	75% (9/12)
Drug trials	73% (8/11)
International research	58% (7/12)

Existence of Standard Operating Procedures	83% (10/12)

Policies to manage conflicts of interest	58% (7/12)

Existence of a 'hot line' to received participants' complaints	50% (6/12)

Research ethics guidelines rated as 'very appropriate'*	
CIOMS	64% (7/11)
Declaration of Helsinki	78% (7/9)
IOMS	56% (5/9)
Belmont Report	14% (1/7)

*Not all of the RECs gave a response to this item

#### Member Composition

The average number of members on the RECs was 10.3 members; median of 9 members and range of 7 to 19 members. All RECs had at least 7 members. Regarding member type, 69.5% of the members from all of the RECs were physicians, 8.5% were pharmacists, 4.8% were scientists, 4.8% were lawyers, and 13.7% were individuals not involved in medical, scientific, or legal work e.g., journalist, ethicist, philosopher, religious leader, and community member). In five RECs, more than 80% of the members were physicians. In 9 RECs, there were members other than someone involved from the medical, scientific, or legal fields. Table 2 shows the numbers of RECs having at least one member of the indicated profession. For example, 92% of the RECs had at least one physician, whereas 50% of the RECs stated they had a community member on their committee.

**Table 2:** Numbers of Research Ethics Committees with at least one of the listed professional type (n = 12)

Membership Category	Category representation on the RECs (% of RECs)
Medical Doctor	92% (11/12)

Pharmacist	42% (5/12)

Non-affiliated Doctor	33% (4/12)

Scientist	17% (2/12)

Nurse	8% (1/12)

Legal Expert	42% (5/12)

Journalist	25% (3/12)

Ethicist	17% (2/12)

Philosopher	8% (1/12)

Religious Leader	8% (1/12)

Administrator	8% (1/12)

Community Member	50% (6/12)

#### Workload

Of the RECs reporting their frequency of meetings (n = 11), 9 stated it met at least once/month, 1 met every 2 months, and 1 stated "other". The average duration of the meetings of these 11 RECs was 2.0 hours (median: 2 hours; range 1-3). Of those who responded (n = 9), RECs reviewed an average of 3.8 protocols per meeting (median: 2.5, range 1 - 10) and an average of 42.3 protocols per year (median: 22; range 1-150). Seven of the RECs reported reviewing 5 or less protocols/meeting. Continuing reviews and expedited reviews were performed by 5 and 3 RECs, respectively.

#### Processes of Research Review

Table 3 shows aspects of the REC review process. Most of the respondent RECs require investigators to use an REC-submission form and most use a primary review system in which all of these RECs attempt to match the subject matter of the protocol to the primary reviewr's expertise. Less than half of the RECs have in place a system whereby the chair or a designated person can approve protocols by an expedited system. Of these RECs, only one has actually used such a system. Of the respondent RECs, 11 of 12 send written notification of its decision to the principal investigator, but only six of these RECs state the expiration date of its approval in the letter. Also, regarding the approval letter, 8 of the RECs required investigators to use the REC-approved informed consent forms, but only 7 of these RECs attach a copy of the consent form to the approval letter. There was another REC that attached its approved consent form to the approval letter, but did not explicitly state in the approval letter the requirement to use it. Only 3 of these RECs stamped the informed consent forms with the expiration date.

**Table 3:** Processes of Ethics Review (n = 12)

Review Process	Response
Investigators are required to submit protocol using an REC submission form	58% (7/12)

A primary review system is used to review protocols	75% (9/12)

Attempt to match subject matter of protocol to primary reviewer's expertise	75% (9/12)

A system is in place whereby the chair or an authorized person is able to approve protocols by an expedited review process	42% (5/12)

Number of days that REC members have to review materials prior to meeting	<3 days (0/11) 3-7 days (4/11) 8-14 days (5/11) >14 days (2/11)

REC notifies investigators in writing of its decision	92% (11/12)

Time interval between meeting and written notification to investigators	Average: 5.2 days Median: 5 Range: 2-10 days

**Contents of Approval Letter**	

For studies approved for 1 year, approval letter states expiration date	50% (6/12)
Requirement to use the REC-approved informed consent form	67% (8/12)
Copy of REC-approved consent form is attached to the approval letter	67% (8/12)
Attached REC-approved consent form is stamped with expiration date	25% (3/12)

#### Perceived Capacity to Review Research and Training

RECs were asked to rate their perceived capacity to review international protocols (capacity was not defined). Of the 11 respondent RECs, 1 reported limited capacity, 3 reported moderate capacities, 6 reported good capacities, and 1 reported it had excellent capacity.

Almost three-quarters (72.6%) of the members from all the 12 RECs indicated they received some training in ethics. Chairs from all of the RECs indicated that they had received some prior ethics training. Respondent RECs indicated that such training consisted of workshops (at least 1/2 day workshop), and that 14.5% of all members had received training from a FIC/NIH training initiative [13]. Eleven RECs indicated that it has at least two members with training in ethics. One REC indicated that none of its members had received any training in research ethics.

Table 4 shows how the respondent RECs rated the importance of a list of training topics using the following categories: 'very important', 'somewhat important', 'not so important', and 'not important'. At least 90% of the RECs considered the following topics to be either "very imporant' or somewhat important': placebo controlled trials; determination of methods to reduce risk; the interpretation of pre-clinical studies; determination of risks in research; assessment of benefits to participants and society; scientific design issues in clinical trials; and monitoring and oversight of approved studies. None of the listed topics was considered as "not so important" or "not important" by more than 3 RECs.

**Table 4:** Topics rated as being "very important" or "quite important".

Topics	Percentage of RECs reporting
The use of placebo controlled trials	100% (10/10)
Determination of methods to reduce risk	92% (11/12)
The interpretation of pre-clinical studies	91% (10/11)
Determination of risks in research	91% (10/11)
Assessment of benefits to participants and society	90% (9/10)
Scientific design issues in clinical trials	90% (9/10)
Monitoring and oversight of approved studies	90% (9/10)
Assessment of cultural sensitivity for informed consent	82% (9/11)
Assessment of understanding of informed consent	80% (8/10)
Access to benefits after the trial is over	78% (7/9)
Determination of appropriate subject selection in vulnerable populations	75% (9/12)
Community participation	67% (6/9)
Social and behavioral studies	75% (9/12)
Privacy and confidentiality	70% (7/10)

### Financial and Material Resources

None of the RECs reported having a budget, while 5 stated that their members receive financial compensation for their activities. Regarding dedicated office space, computer equipment, and secretarial support, 50% (6/12), 58% (7/12), and 50% (6/12) of the RECs, respectively, indicated that such resources were available to their committees. Other RECs reported having shared institutional access to these resources. Finally, of the 12 RECs, 8 stated that its chair performs administrative duties and 6 RECs reported using a computerized database to track protocols.

### Challenges to Functioning

Table 5 shows the perceived challenges to REC functioning. Most of the reported challenges relate to a lack of guidance from the national level in terms of research ethics guidelines, standards for the operation of ethics committees, and an accreditation mechanism for RECs. Other frequently mentioned challenges involve the inability to monitor approved protocols, lack of ongoing training for its members, and insufficient member competence to review protocols.

**Table 5:** Challenges to proper functioning of the research ethics committees

Challenges	Frequency of RECs reporting challenge
The need to develop appropriate national ethics guidelines	92% (11/12)
Inadequate ability to monitor approved protocols	91% (10/11)
Lack of ongoing training for members in research ethics	82% (9/11)
Lack of national accreditation mechanism for ethics committees	67% (8/12)
Lack of national standards for operation of committees	58% (7/12)
Competence of members to review research protocols	55% (6/11)
Variable use of ethical guidelines across committees in Egypt	33% (4/12)
Lack of coordination between different committee	33% (4/12)
Difficulties adapting international guidelines to local conditions	25% (3/12)

## Discussion

This survey of existing RECs in Egypt has identified important operating structures, review processes, workloads, and financial and material resources of these committees. All of these characteristics can be considered as important proxies for REC effectiveness, defined as achieving the mandate to protect research participants. This survey also shows that variability exists among the respondent RECs regarding their functional characteristics.

Specifically, our survey shows that many of the RECs have enabling characteristics needed for effective functioning [4,14-17]. For example, regarding operational structures and processes, most of the surveyed RECs have SOPs and many have conflict of interest policies. Also, all RECs report to high institutional officials, indicating that their mandate comes from a high authority that legitimizes its existence to their peers. It appears that many are active, as most of the RECs (9/11) reported meeting at least once a month and are actively reviewing protocols. The average duration of their meetings (2 hours) appears appropriate to the average number of protocols reviewed at each meeting (3.8 protocols/meeting). Most of the RECs use international research ethics guidelines in their review of protocols and these included CIOMS, IOMS, and the Declaration of Helsinki. In contrast, the Belmont Report is used sparingly, which is consistent with surveys of RECs from other developing regions [10,11]. This finding probably reflects that while the Belmont Report serves as an important source of a principled approach to the conduct of research in general, its specification of the principles of research ethics is limited in its scope and relevance for international research.

Finally, many RECs adopt review processes recommended by many authorities [14,17]. These processes include sufficient time in advance of the meeting for members to review the relevant documents, use of a primary review mechanism, attempts to match subject matter of the protocol to the primary reviewer's expertise, and notification of investigators of its decisions in a timely fashion and requirement that REC-approved consent forms be used in the research. Finally, the number of REC members with prior ethics training (approximately 75% of the current membership and 100% of the chairs) is higher than that reported in previous surveys of RECs in other developing countries [10,18]. To be sure, the target goal of member training should be 100% to ensure that "members have knowledge, skills, and abilities appropriate to their respective roles" [19].

Our survey does demonstrate areas for improvement. First, while our study indicates that most committees exceed current minimum membership requirements in terms of numbers, the diversity of membership appears to be inadequate. Specifically, our survey showed that there is a dominance of physicians/scientists on the existing RECs, as close to 88% of the members of the RECs represented the scientific/medical/legal community. This level exceeds international membership trends; for example, a survey of 89 RECs in the United States in 2001 found that physicians, scientists and pharmacists together made up 46% of REC membership [20]. Our survey results regarding member composition is similar to what has been reported in other developing regions. For example, in a survey consisting of 12 RECs in South Africa, doctors, scientists, and pharmacists together made up 61% of the membership [11]. In another survey of RECs in Southern Africa that reviewed HIV vaccine trials, doctors, scientists, and nurses comprised 67% of the membership [10].

Regarding members other than those in the medical, scientific, or legal fields, only 9 RECs in our survey had at least one of this member type and 5 of these RECs had only one such member. Many guidelines recommend an adequate diversity of membership. The Department of Health in South Africa issued national guidelines that require RECs to be "representative of the communities they serve and increasingly reflect the demographic profile of the population of South Africa... [and that there must be] "at least two lay persons with no affiliations with the institution, not currently involved in medical, scientific or legal work" [21]. The National Bioethics Advisory Commission in the United States recommends that non-scientists make up at least 25% of an REC membership [22] and the United Kingdom requires that one third of the REC membership be community members [23]. Membership diversity is considered important in carrying out the REC charge of participant protection, as community members will be more knowledgeable and sensitive to the concerns of those who participate in clinical research. Critics have claimed that an imbalance in membership in favor of researchers and other institutional members biases the process towards the interests of researchers versus the interest of research participants [24]. Adding to the potential bias is that chairs are often institutional scientists. Also, an REC that has a large proportion of the membership consisting of affiliated scientists/clinicians might hinder an objective and fair discussion of the protocol being reviewed, as unaffiliated members from the community might feel intimidated from existing power differentials between themselves and scientists/clinicians [24].

Having said this, a distinction needs to be made between lay and community representation. Lay representation often refers to individuals with no scientific or medical background and hence, could include lawyers, ethicists, priests, or theologians who have higher levels of education than individuals from the communities being researched and hence, might not be able to assess the research from the perspective of those who actually participate in the research. Community representatives, on the other hand, would refer to non-professional, non-scientific members who belong to the community that is being researched and would more likely reflect the culture and values of the involved community [11]. These issues of adequate community representation, however, can often be clouded by ambiguity regarding how to define the actual community, as well as who can serve as the legitimate representatives of the communities.

Finally, many RECs reported that they lack essential financial and capital resources thought to be essential for a well-functioning committee. For example, all of the RECs operated without a budget and many were without important dedicated office, computer, and secretarial support. Also, three-quarters of the chairs were reported to having to perform administrative duties. These findings regarding financial and material resources are similar to those reported by RECs in South Africa [10,11].

RECs' challenges to effective functioning included a lack of guidance at the national level. For example, they cited the absence of national research ethics guidelines, as well as the lack of national standards for RECs. Other frequently mentioned challenges included lack of ongoing training for its members, inability to monitor approved protocols, and lack of a national accreditation mechanism for ethics committees.

Many RECs also reported topics believed important for their functioning and included placebo-controlled trials, scientific design issues in clinical trials, determination of risks and minimization of risks, assessment of benefits, monitoring and oversight of approved studies, and informed consent issues. The type of issues considered important are similar to those reported by RECs in Southern Africa [10,11].

Finally, it is important to note that our survey shows variability among the respondent RECs in many of the structural and operating processes, including member composition, existence of written SOPs and conflict of interest policies, access to adequate financial and material resources, and processes of protocol review. The existence of variability in structure and processes probably equates to variability in functioning between the different RECs that might not offer a consistent protection mechanism for research participants across the country. These results strongly advocates the promotion of national operating standards for RECs, as well as the establishment of monitoring and oversight mechanisms for RECs, including the institution of an accreditation process for RECs. As a start, guidance can be given in the form of a self-assessment tool that RECs can use to gauge its own level of functioning.

There are several limitations to our study. First, the responses were based on a process of self-report and accordingly, there might have been a tendency to over report the achievements of individual RECs as well as underreport weaknesses. Second, the failure of other existing RECs to complete the survey might represent an element of bias to our aggregate results. Also, our results for the 'high' level of members trained in research ethics might be biased by the manner in which we identified the RECs. For example, we identified REC members who had attended research ethics workshops or those who had attended a Fogarty-sponsored intensive training course in research ethics. Another limitation of our survey was that we failed to inquire about other types of information. For example, we neglected to inquire about the gender composition of the individual RECs, as well as information important in identifying challenges to the independence of RECs. Finally, our results might not be generalizable to other countries in the Middle East.

Notwithstanding these limitations, our survey documents several areas of strengths and areas for improvements in the operations of RECs in Egypt. Regarding strengths, many of the RECs meet frequently, have a majority of members with prior training in research ethics, and have written policies, including those on SOPs and conflicts of interest. Regarding areas for improvements, many RECs should strive for a more diverse membership and should receive more financial resources and administrative support personnel. Finally, most RECs agree that the absence of national ethics guidelines and ongoing training of their members represent challenges for their functioning.

## Conclusions

Our general recommendations include that RECs should solicit individuals from the community to become members and develop a continuing educational program for its members. Institutional officials should be aware of the resource capacity needs of the RECs in Egypt. Dialogue should begin regarding the development of national research ethics guidelines, national operating standards for RECs, and the establishment of an accreditation mechanism for RECs that can reduce variability among the existing RECs.

## Abbreviations

RECs: research ethics committees; OHRP: Office of Human Research Protections; SOPs: standard operating procedures; FIC/NIH: Fogarty International Center/National Institutes of Health; NGO: non-governmental organization; HIV: Human Immunodeficiency Virus; CIOMS: Council for International Organizations of Medical Sciences; IOMS: Islamic Organization for Medical Sciences.

## Competing interests

The authors declare that they have no competing interests.

## Authors' contributions

HS helped conceived the idea of the study, contributed to the design of the survey tool and its distribution, helped with the analysis of the data and with the writing of the manuscript.

SEK contributed to the design of the survey tool and its distribution and the analysis of the data.

HJS helped conceived the idea of the study, contributed to the design of the survey tool and its distribution, helped with the analysis of the data and with the writing of the manuscript.

All authors read and approved the final manuscript.

## Authors' information

HS is chair of the Research Ethics Committee at the National Hepatology and Tropical Medicine Research Institute, Cairo, Egypt

SEK is Assistant Professor in the Department of Epidemiology and Preventive Medicine, University of Maryland School of Medicine.

HJS is Professor of Medicine in the Department of Medicine, University of Maryland School of Medicine and Program Director of the Middle East Research Ethics Training Initiative (MERETI).

## Pre-publication history

The pre-publication history for this paper can be accessed here:

http://www.biomedcentral.com/1472-6939/11/12/prepub
